# Modeling socio-demographic and clinical factors influencing psychiatric inpatient service use: a comparison of models for zero-Inflated and overdispersed count data

**DOI:** 10.1186/s12874-020-01112-w

**Published:** 2020-09-16

**Authors:** Sharmin Sharker, Lloyd Balbuena, Gene Marcoux, Cindy Xin Feng

**Affiliations:** 1 School of Public Health, University of Saskatchewan, 104 Clinic Place, Saskatoon, Canada; 2 Department of Psychiatry, College of Medicine, University of Saskatchewan, 103 Hospital Drive, Saskatoon, S7N 0W8 Canada; 3 Department of Community Health and Epidemiology, Faculty of Medicine, Dalhousie University, 5790 University Avenue, Halifax, B3H 1V7 Canada

**Keywords:** Repeated hospitalizations, Zero-inflated models, Hurdle models, Model comparison, Model diagnosis

## Abstract

**Background:**

Psychiatric disorders may occur as a single episode or be persistent and relapsing, sometimes leading to suicidal behaviours. The exact causes of psychiatric disorders are hard to determine but easy access to health care services can help to reduce their severity. The aim of this study was to investigate the factors associated with repeated hospitalizations among the patients with psychiatric illness, which may help the policy makers to target the high-risk groups in a more focused manner.

**Methods:**

A large linked administrative database consisting of 200,537 patients with psychiatric diagnosis in the years of 2008-2012 was used in this analysis. Various counts regression models including zero-inflated and hurdle models were considered for analyzing the hospitalization rate among patients with psychiatric disorders within three months follow-up since their index visit dates. The covariates for this study consisted of socio-demographic and clinical characteristics of the patients.

**Results:**

The results show that the odds of hospitalization are significantly higher among registered Indians, male patients and younger patients. Hospitalization rate depends on the patients’ disease types. Having previously visited a general physician served a protective role for psychiatric hospitalization during the study period. Patients who had seen an outpatient psychiatrist were more likely to have a higher number of psychiatric hospitalizations. This may indicate that psychiatrists tend to see patients with more severe illnesses, who require hospital-based care for managing their illness.

**Conclusions:**

Providing easier access to registered Indian people and youth may reduce the need for hospital-based care. Patients with mental health conditions may benefit from greater and more timely access to primary care.

## Background

The increased demand for health care for mental health concerns has been identified as an important public health topic in many countries. Repeated inpatient hospitalizations within a short period may be a reflection of not only the quality of the hospital care [1] but also an expensive mode of treatment [2–4].

Elucidating factors that contribute to repeated inpatient mental health hospitalizations is essential for understanding the population heterogeneity of mental health care seeking behaviors. The frequent use of inpatient mental health care may be attributable to the underlying lack of access to outpatient care [5] and substandard hospital care [6, 7]. For example, a recent study conducted in Canada reported that having a good connection to a primary care provider decreased the probability of being a high-cost health service user [8]. Inpatient hospitalizations for patients with psychiatric disorders were reported that depend on the type of illness; that is, inpatient readmission is common for individuals with severe mental illness (e.g., schizophrenia, mood disorders, bipolar disorder and psychoses) [9–13]. Demographic and socio-economic factors have been shown to be associated with mental health services utilization. Recent studies indicated that mental health problems among children and youth have been increasing over the last three decades [14, 15]. For example, the prevalence rates of mental disorders among children and youth in Canada was estimated to be about 14% [16]. A survey conducted in Ontario [17] showed that about one in five children between the ages of 4 and 16 years experience at least one of the following psychiatric disorders (conduct disorder, hyperactivity, emotional disorder, and somatization). Carriere et al. [18] found that hospitalization rates for mental illness were higher for Aboriginals living on and off reserve, Metis, and Inuit than for the non-Aboriginal population, regardless of disease category. Females were found to have higher use of health facilities for psychiatric illness than males [8]. Geographical characteristics such as population density, place of residence and proximity to service have been identified as important factors in several studies. Some studies reported that readmission rates were lower in urban regions [19, 20]; whereas, positive associations between readmission rates and population density were reported in other studies [19].

In order to model hospitalization rates (i.e. number of hospitalizations per unit time), many studies have focused on the population with at least one hospitalization admission, neglecting people with no hospitalizations in a given period. Priebe [21] considered the readmission rate per person-year; while in other cases, separate analyses were performed for psychiatric and non-psychiatric reasons [22]. Various comparisons involved patients readmitted during a given time period versus a control group of non-readmitted patients [23], early versus late readmission versus control patients [24, 25], or readmitted versus several groups of non-readmitted [26] (community and nursing home). Several studies simply compared those patients who have been readmitted versus not readmitted–ignoring number of readmissions. The latter could lead to the loss of pertinent information, such as the distribution of readmissions.

To address these gaps, an electronic prospective cohort was created that included patients who had not experienced any psychiatric hospitalizations. Our objective was to identify socio-demographic and clinical factors associated with repeated hospitalizations by using count regression models accounting for zero-inflation and overdispersion, and contribute to the literature in mental health care utilization in Canada.

## Methods

### Setting

Saskatchewan is a landlocked province in central Canada and is bordered by Alberta in the west and Manitoba in the east. Saskatchewan is also a primarily agricultural province. The population is concentrated in the two largest cities of Saskatoon and Regina. Saskatchewan is the birthplace of Medicare, or universal health coverage [27]. With universal health coverage, residents, out-of-province Canadians, immigrants, foreigners with a work visa, international students are normally covered for doctor’s visits and hospitalizations at most three months after arrival [28]. Prescription medications and dental services are usually not covered.

### Study design

An electronic cohort of psychiatric patients who did not have a previous hospitalization for mental health conditions was constructed. Subjects entered the study if they had a physician services claim in the Medical Services Plan database between January 1, 2010 and December 31, 2011 reporting a psychiatric diagnosis, i.e., International Classification of Diseases (ICD)-9: 290-319. The index date is the date of the first psychiatric service record during these two years. All patients were followed up for 3 months after the index date. The exit date is the earliest of December 31, 2012, death, or coverage termination with the Saskatchewan Ministry of Health. To allow for a sufficient follow-up, a minimum of 30 days follow-up period was considered. Past (January 1, 2008 to December 31, 2009) and future (January 1, 2010-December 31, 2012) hospital separation records were then extracted for cohort members. A wash-out period of two years prior to the index date was implemented to exclude participants who had hospitalizations due to any psychiatric illness during this period. Excluding these people left us with a non-hospitalized group whose hospitalization would reflect a worsening of their condition. With a very low incidence of psychiatric disorders in children with ages (0-5) years (1.4%), we decided to exclude them from the analysis. Then we categorized the remaining individuals aged between 6 to 86 into quartiles.

### Data sources and description

#### Data source

We used Saskatchewan administrative health databases to identify our study population of patients. To obtain socio-demographic data, disease type and service use, we linked the hospital separations (admission date, discharge date, diagnosis codes and diagnosis type) to the person registry database (gender, year of birth, study entry date, study index date, study exit date, reason for exit, their registered Indian status and residence at index date), and the medical service database (date of visit and diagnosis) and physician mobility database (medical speciality). The data was provided by Saskatchewan’s Ministry of Health. A brief description of the databases is given below:

***Person registry database***: Each patient was assigned a study ID number. The person registry consists of the patient’s gender, year of birth, study entry date, study index date, study exit date, reason for exit, their registered Indian status and the regions of residence at the index date, which were categorized into four categories: (1) Regina census metropolitan area, (2) Saskatoon census metropolitan area, (3) Lloydminster, Moose Jaw, North Battleford, Prince Albert, Swift Current, Yorkton, and (4) rest of Saskatchewan.

***Hospital separation database***: The hospital separation database contains information about admission date, discharge date, diagnosis codes and diagnosis type.

***Physician services database***: The physician services database includes date of visit, diagnosis, doctor ID and referring doctor ID. Services delivered to a particular person by a particular physician for the same diagnosis on the same day at the same clinic are reduced to a single visit record.

***Physician mobility database***: This database contains the specialty of a particular physician or medical care provider for the purpose of fee-for-service payment rates.

#### Outcome variable

The outcome of interest in this study is the number and the odds of hospitalizations among the patients with psychiatric disorders within the first 3 months of their index dates. The diagnosis codes for hospitalizations were collected from the Hospital separation databases, where there were 25 diagnosis codes and diagnosis types listed for each person and each visit. We considered the very first diagnosis code and the first diagnosis type as the indicator of disease type for each patient.

#### Predictors of hospitalizations

Clinical variables consisted of psychiatric diagnoses measured on the index date that were grouped into: schizophrenia, anxiety, behavioural disorders, mood disorders, substance use and others. Table 1 lists the specific ICD codes for each category. Clinical variables also included outpatient visits to fee-for-service (FFS) psychiatrists and general physician (GP) for a mental health condition that occurred within two years before the index visit. We cross-referenced patient hospitalizations with GP and FFS psychiatrist visits by looking up the doctors’ speciality in the physician mobility database. Socio-demographic variables included age groups (6-29, 29-45, 45-60 and 60-86 years of old) registered Indian status (ever/never), gender (male/female) and location of residence measured on the index date including Saskatoon, Regina, Lloydminster or others (MooseJaw, North Battleford, Prince Albert, Swift Current, Yorkton) and rest of Saskatchewan.

**Table 1:** Categorization of the Psychiatric Diagnoses

**Categories**	**Psychiatric Diagnoses**
Schizophrenia	Simple/acute type of schizophrenia, Paranoid states
(ICD code: 295, 297, 298)	
Anxiety	Phobic states, Neurotic depressive states,
(ICD code: 300,308, 309)	Obsessive-compulsive disorders
Behavioral disorder	Autism, psychoses with origin specific to childhood,
(ICD code: 299, 312-315)	Disturbance of emotions specific to childhood and adolescence.
Mood disorder	Manic-depressive psychosis, Any kind of depressive disorder
(ICD code: 296, 311)	
Substance use	Alcoholic/drug psychosis, Paranoid or hallucinatory states induced by drugs,
(ICD code: 291, 292, 303)	Dependent/Non-dependent tobacco/cocaine use disorder.
Others	Dementia, Acute confusional state, Non-alcoholic psychosis,
(ICD code: 290, 293, 294)	Gender deviations disorders, Predominant disturbance of emotions,
	Cyclical vomiting/sleep disorder/hair plucking.

### Statistical methods

The longitudinal data included 200,537 patients with psychiatric disorders from Jauary 1, 2008 to December 31, 2012. The number of hospitalizations in our data was heavily right-skewed with a large number of zeros, i.e, 199,271 (99.36%). For modeling counts data, the choice of underlying distribution is crucial for valid statistical inference. Poisson regression is commonly used for analyzing counts data [29–32], but it requires the mean and variance to be equal conditional on a given set of covariate values, [33–35]. Poisson regression may not perform well when this requirement is not met [36], a condition known as overdispersion. The negative binomial (NB) distribution is often used with overdispersed data [37] but neither Poisson or NB regressions may fit the data well [38].

To accommodate the excess zeros, hurdle and zero-inflated models are often used. A hurdle model [39] is a two-component model in which one component models the probability of zero counts and the other component uses a zero-truncated Poisson or zero-truncated NB distribution that is conditional on a positive outcome. Another way to deal with excessive zeros is a zero-inflated model [40], which is a mixture of a regular count regression model such as Poisson or NB model and a component that accommodates the excessive zeros. Given the nature of our data, we applied and compared zero-inflated Poisson (ZIP), zero-inflated negative binomial (ZINB), hurdle Poisson (HP), hurdle negative binomial (HNB) and the conventional count regression models i.e. Poisson and NB models in this analysis.

Models were assessed using Akaike’s Information Criterion (AIC) [41] and Vuong’s test [42]. AIC is defined as AIC= -2log$L(\hat {\theta })$+2c, where $L(\hat {\theta })$ is the maximized likelihood function of a candidate model given the data when evaluated at the maximum likelihood estimate of *θ* and $-\text {log} L(\hat {\theta })$ offers summary information on how much discrepancy exists between the candidate model and the data, where *c* is the number of estimated parameters in the candidate model. A lower AIC indicates a better fit of the model to the data. Vuong’s test [42] is a likelihood-ratio-based test for model comparison in which the null hypothesis sets the two models equal to one another. The test statistic is given by $V= \frac {\bar {m}\sqrt {n}}{S_{m}}$ with $m_{i}=\text {log}\left [\frac {\hat {P_{1}}\left (Y_{i}|X_{i}\right)}{\hat {P_{2}}\left (Y_{i}|X_{i}\right)}\right ]$, where *m*_*i*_ is the log-likelihood ratio between two models with $\hat {P_{1}}\left (Y_{i}|X_{i}\right)$ and $\hat {P_{2}}\left (Y_{i}|X_{i}\right)$ denoting the likelihood of two models. The statistic *m*_*i*_ has a mean $\bar {m}$ and standard deviation *S*_*m*_ and *n* is the sample size. The statistic *V* asymptotically follows a standard normal distribution. *V* greater than 1.96 supports $\hat {P_{1}}\left (Y_{i}|X_{i}\right)$ and *V* less than -1.96 supports the $\hat {P_{2}}\left (Y_{i}|X_{i}\right)$ at 5% level of significance.

For model diagnosis, randomized quantile residual (RQR) [43] is used, which is particularly useful to diagnose the models for modelling discrete and skewed data. The key idea is to invert the fitted distribution function for each response value and find the equivalent standard normal quantile. Under a correctly specified model, RQRs are approximately normally distributed and the plot of RQRs against the predicted values should be randomly scattered without any discernible pattern [44]. Further, a well-fitting regression model results in predicted values of the outcome variable close to the observed data values [45, 46]. We therefore compared the predicted vs. observed number of psychiatric inpatient visits for all the competing models.

We also conducted sensitivity analyses based on other follow-up time frames defined as 6 and 9 months after the index discharge to check the consistency of results over all the study periods. We focus on presenting the results for 3 months study period, as the results are consistent for all three study periods. Statistical analyses were performed using R version 3.4.1 (R Foundation for Statistical Computing, Vienna, Austria) with the glmmTMB package [47].

## Results

### Descriptive analysis

Table 2 presents the descriptive statistics of patients’ clinical and social-demographic profiles. Of 200,537 cohort members, only 1266 were hospitalized and 199,271 were not hospitalized within three months. The highest number of patients who were hospitalized were those who had behavioral disorder (5.38%) as the primary diagnosis at the index visit.

**Table 2:** Descriptive statistics for the number of inpatient hospitalizations

**Variables**	**Yes(n=1266)**	**No(n=199271)**
**Clinical characteristics**		
Disease category		
Schizophrenia	209(1.67%)	12364(98.33%)
Anxiety	334(0.41%)	81840(99.59%)
Behavioral disorder	71(5.38%)	12517(94.62%)
Mood disorder	481(0.79%)	60223(99.21%)
Substance use	110(0.62%)	17833(99.38%)
Others	61(0.42%)	14494(99.58%)
Outpatient FFS Psychiatrist visit		
Yes	311(1.92%)	15920(98.08%)
No	955(0.52%)	183351(99.48%)
Outpatient GP visit		
Yes	952(0.52%)	179788(99.48%)
No	314(0.58%)	19483(99.42%)
**Social-demographic characteristics**		
Registered Indian		
Ever	217(0.99%)	21596(99.01%)
Never	1049(0.59%)	177675(99.41%)
Gender		
Male	586(0.71%)	81478(99.29%)
Female	680(0.58%)	117793(99.42%)
Age		
(6-29]	466(0.90%)	51401(99.10%)
(29-45]	268(0.53%)	49931(99.47%)
(45-60]	226(0.46%)	49303(99.54%)
(60-86]	306(0.63%)	48636(99.37%)
Residence		
Saskatoon	239(0.42%)	56860(99.58%)
Regina	241(0.57%)	41931(99.43%)
Lloydminster or others ^*a*^	188(0.67%)	28239(99.33%)
Rest of Saskatchewan	598(0.83%)	72241(99.17%)

a= MooseJaw, North Battleford, Prince Albert, Swift Current, Yorkton

Table 3 reports the results of model comparison based on the AIC and Vuong’s test scores, which shows that HNB had the lowest AIC and −2 log-likelihood. The result of Vuong’s test also indicated HNB outperformed Poisson, NB, ZIP and HP models, as it yielded the Vuong’s test score lower than -1.96. Although this test did not show much difference between the performance of ZINB and HNB, the results of AIC and −2 log-likelihood indicated that HNB outperformed the other models. For model diagnosis, the normal quantile-quantile (QQ) plots (Figure S1 in the supplementary materials) showed that the RQRs under HNB model more closely aligned along the diagonal line as compared to other competing models. The scatter plot of RQRs (Figure S2 in the supplementary materials) showed that no discernible pattern in various models. However, only RQRs with the HNB are bounded between -4 to 4. Table S1 in the supplementary materials presented the observed vs. predicted counts of hospitalizations which showed that the prediction under the HNB model is more precise in comparison to other models.

**Table 3:** Model comparison based on AIC, -2 Log likelihood and Vuong’s test for the competing models (*p*-values for Vuong’s test are given in the parentheses)

**Model**	**AIC**	**-2 Log likelihood**	**Vuong’s test**				
			**NB**	**ZIP**	**ZINB**	**HP**	**HNB**
Poisson	16839	16807	-5.812	-6.164	-6.035	-6.004	-5.958
			(<0.001)	(<0.001)	(<0.001)	(0.009)	(<0.001)
NB	15689	15655		-1.477	-4.852	-0.764	-4.236
				(<0.069)	(<0.001)	(0.222)	(<0.001)
ZIP	15664	15590			-3.823	2.346	-3.269
					(<0.001)	(0.009)	(<0.001)
ZINB	15572	15496				4.615	-0.178
						(<0.001)	(0.4292)
HP	15644	15570					-4.444
							(<0.001)
**HNB**	15534	15458					

Table 4 presents the estimated regression coefficients of the best fitting model, i.e., HNB model. The results consist of two separate parts: one models the odds of being hospitalized and the other pertains to the number of hospitalizations for those who had at least one hospitalization. The logistic component of HNB showed that registered Indians had 1.59 (95% CI: 1.36, 1.86) times higher odds of being hospitalized than non-registered Indians. Patients who visited GP prior to index dates had 0.63 (95% CI: 0.53, 0.75) times lower odds of being hospitalized than those patients who did not visit GP for psychiatric concerns. Patients from Lloydminster, Regina and the rest of Saskatchewan were respectively 1.93 (95% CI: 1.58, 2.34), 1.64 (95% CI: 1.37, 1.97) and 2.38 (95% CI: 2.03, 2.78) times more likely to be hospitalized than patients from Saskatoon. The odds of getting hospitalized for the patients aged between 6 to 29 years and 30 to 45 years old were 2.15 (95% CI: 1.81, 2.56) and 1.22 (95%CI: 1.01, 1.46) times higher than those who are aged between 61 to 86. Males were 1.22 (95% CI: 1.09, 1.37) times more likely to be hospitalized than females. The results of the count component of the HNB model indicated that the patients who had previously visited any FFS psychiatrist have 1.99 (95% CI: 1.33, 2.97) times higher risk of repeated or multiple hospitalizations. Patients from Regina were 0.37 (95% CI: 0.20, 0.68) times less likely to have repeated hospital readmissions than patients from Saskatoon.

**Table 4:** Parameter estimates of the best-fitting model (HNB model) for the 3 months follow up period (n=200,537). OR: odds ratio, RR: risk ratio

**logit[*P*(*Y***_***i***_**>0)]**	**OR**	**95% CI of OR**		***P*-value**
		**Lower**	**Upper**	
Registered Indian (Yes vs. No)	1.59	1.36	1.86	<0.001*
GP visit (Yes vs. No)	0.63	0.53	0.75	<0.001*
Lloydminster vs. Saskatoon	1.93	1.58	2.34	<0.001*
Regina vs. Saskatoon	1.64	1.37	1.97	<0.001*
Rest of Saskatchewan vs. Saskatoon	2.38	2.03	2.78	<0.001*
Age [6,29] vs. Age [61,86]	2.15	1.81	2.56	<0.001*
Age [30,45] vs. Age [61,86]	1.22	1.01	1.46	0.041*
Age [46,60] vs. Age [61,86]	0.99	0.82	1.20	0.941
Gender (Male vs. Female)	1.22	1.09	1.37	<0.001*
log[ *E*(*Y*_*i*_|*Y*_*i*_>0)]	RR	95% CI of RR		*P*-value
		Lower	Upper	
Psychiatric visit (Yes vs. No)	1.99	1.33	2.97	<0.001*
Lloydminster vs. Saskatoon	0.58	0.31	1.08	0.090
Regina vs. Saskatoon	0.37	0.20	0.68	0.001*
Rest of Saskatchewan vs. Saskatoon	0.84	0.53	1.31	0.444
Age [6,29] vs. Age [61,86]	0.61	0.38	1.00	0.050*
Age [30,45] vs. Age [61,86]	1.04	0.63	1.71	0.862
Age [46,60] vs. Age [61,86]	1.18	0.70	1.97	0.521
Anxiety vs. Others	1.07	0.41	2.76	0.888
Behavioral disorder vs. Others	1.43	0.43	4.69	0.548
Substance use vs.Others	1.11	0.37	3.28	0.849
Mood disorder vs. Others	2.11	0.85	5.20	0.102
Schizophrenia vs. Others	1.27	0.48	3.38	0.621

Psychiatric visit: Outpatient Psychiatric visit prior to index date.

GP visit: Outpatient GP visit prior to index date

Outpatient psychiatric visits and disease categories had an interacting effect for the propensity of being hospitalized based on the results of the logistic component of the HNB model, as shown in Table 5. Among patients who had visited a psychiatrist, those who primarily suffered from Schizophrenia were 1.812 (95% CI: 1.080, 3.039) times more likely to be hospitalized during the follow-up compared with those patients with disorders grouped in the “others” category. By comparison, among the patients who did not visit any psychiatrist previously, those who had Schizophrenia as the primary diagnosis had a much higher risk of being hospitalized later on, i.e., 6.112 (95% CI: 4.232, 8.826) times more likely to be hospitalized compared with those in the “others” category. This implied that previous visits to a psychiatrist may play a protective role against hospitalization for patients with Schizophrenia compared with those in the “others” category.

**Table 5:** Odds ratio (OR) and 95% CI for the interaction effects between disease category and FFS psychiatric visits for the logistic component of the HNB model

**Psychiatric visits**	**Disease category**	**OR**		
		**Estimate**	**95% CI**	
			**Lower**	**Upper**
Yes	Anxiety vs. Others	0.608	0.359	1.031
	Behavioral disorder vs. Others	0.385	0.220	0.673
	Substance use vs. Others	1.123	0.595	2.120
	Mood disorder vs. Others	0.888	0.548	1.439
	Schizophrenia vs. Others	1.812	1.080	3.039
No	Anxiety vs. Others	1.459	1.041	2.044
	Behavioral disorder vs. Others	0.771	0.485	1.225
	Substance use vs. Others	1.586	1.083	2.323
	Mood disorder vs. Others	2.706	1.938	3.777
	Schizophrenia vs. Others	6.112	4.232	8.826
Yes vs. No	Anxiety	3.105	2.245	4.294
	Behavioral disorder	3.719	2.310	5.989
	Substance use	5.272	3.192	8.707
	Mood disorder	2.444	1.915	3.118
	Schizophrenia	2.208	1.586	3.074
	Others	7.448	4.271	12.988

Among patients who visited outpatient psychiatrists previously, there was no difference in getting hospitalized between patients with anxiety, mental disorders due to substance use and mood disorders compared to “other” category. For the patients who did not have previous visits to any FFS psychiatrist and who suffered from anxiety, mental disorder due to substance use and mood disorder had higher odds, i.e., respectively 1.459 (95% CI: 1.041, 2.044), 1.586 (95% CI: 1.083, 2.323), 2.706 (95% CI: 1.938, 3.777) times more likely to be hospitalized compared to those in the “others” category of diseases. Patients who had behavioral disorder had 0.771 (95% CI: 0.485, 1.225) times lower odds than the patients in the “others” category, but not statistically significant. Among the patients in the “others” category, those who had previous outpatient psychiatrist visits (vs. none) had the highest odds of being hospitalized OR: 7.448 (95% CI: 4.271, 12.988). For those who had substance related disorders, the odds of being hospitalized were 5.272 (95% CI: 3.192, 8.707) times higher for those who saw an outpatient psychiatrist vs. not. Similar findings were observed for other disease categories, i.e., behaviour disorder, anxiety, mood disorder and schizophrenia. Interestingly, the HNB counts component, showed that disease category was not associated with the hospitalization count, which highlighted the limitation of only considering the population with at least one hospitalization admission, neglecting people with no hospitalization.

## Discussion

In this study, various counts models including Poisson, negative binomial, zero-inflated Poisson, zero-inflated negative binomial, hurdle Poisson, hurdle negative binomial were considered for analyzing inpatient hospitalization data. We fit each of these models to linked administrative health data, in which the outcome variable was the count of repeated hospitalizations for psychiatric conditions. The negative binomial model fit the data much better than the Poisson model based on AIC, Vuong’s test, and randomized quantile residuals. Furthermore, the hurdle negative binomial model provided the best fit. In the present study, all the study participants had at least one diagnosis of mental health condition and therefore are at risk of being hospitalized. This small risk for the majority of patients and the repeated visits of a few patients are more adequately modeled by techniques that take both zero inflation and count outcomes into account.

This study leads to a better understanding of factors contributing to increased inpatient hospitalizations among patients with mental health conditions. Our results indicated that the odds of having at least one hospitalization vs. no hospitalization for mental disorders were significantly higher among Aboriginal than non-Aboriginal people, but no significant difference was detected in the hospital readmission rate between Aboriginal vs. non-Aboriginal people based on the conditional counts component in the HNB models. HNB models may provide a superior fit to data that reflect a threshold and a counting process that are distinct. Previous literature suggested a number of factors which may contribute to the higher hospitalization rates for mental or behavioural disorders among Aboriginal people. Those factors include traumatic impacts of the residential school and colonization, which may have placed registered Indian people at a higher risk of mental illnesses such as depression and psychological distress [48, 49]. Inequalities in the social determinants of health may also influence hospitalization rate disparities, such as limited educational and employment opportunities and having low income can also lead to difficulties for registered Indian people seeking primary health care [48, 50]. It is also possible that they may encounter barriers when seeking primary health care [51–53] or perceive discrimination as patients [50].

Over the past decade, the prevalence of mental health diagnoses has been rising among young patients seeking acute medical care [54]. A recent comprehensive review of the field of child psychiatric epidemiology [55] noted that the number of observations with mental health issues in community surveys of children and adolescents has risen from 10,000 in studies published between 1980 and 1993 to nearly 40,000 from 21 studies published between 1993 and 2002 [56]. The results of these studies indicate that about one out of every three to four youths is estimated to meet lifetime criteria for a Diagnostic and Statistical Manual of Mental Disorders (DSM) mental disorder [55]. However, a small proportion of these youth actually have sufficiently severe distress or impairment to warrant intervention [57]. According to the Substance Abuse and Mental Health Services Administration, about 1 in 10 youths have a serious emotional disturbance [56, 57]. Our results support this finding, as the logistic component of the HNB model indicated that age was negatively associated with propensity of hospitalization, i.e., younger people aged from 6 to 29 had a higher likelihood being hospitalized for mental health concerns (Table 4). Nevertheless, as shown in the results for the counts components of the HNB models, no significant association between the number of repeated hospitalizations and age was identified for those patients who had at least one hospitalization over the study period for mental health concerns. We speculate that younger people are more likely to be hospitalized for urgent help for mental illnesses, which might imply that young people who were dealing with serious anxiety or depression had lack of access to counseling services or outpatient FFS psychiatric care. This suggests that younger population are a priority population for the development of a standard approach to ensure adequate resources for this population with mental health conditions.

According to World Health Organization (WHO) [58], sex/gender differences are common in the rates of common mental disorders, including depression, anxiety and somatic complaints. These disorders, which have higher prevalence among women, affect approximately one third people in the community and constitute a serious public health problem. Some studies reported that although females have a higher prevalence rate, burden of illness, and likelihood of seeking outpatient treatment for psychiatric disorders; they are less likely than males to receive formal mental health care services, and more likely to receive pharmacological prescriptions from primary care providers [59–61]. Some of the possible reasons of the gender differences in access to mental health care may be because of women’s autonomy, child bearing responsibilities or health literacy regarding psychiatric illness. Our results based on the logistic regression part of the HNB model indicate that males are more likely to be hospitalized. This result is consistent over the three study periods. On the other hand, for the counts regression component of the HNB model, gender did not play any significant role over three follow-up periods. Further investigation is needed to understand the inconsistency of our finding with the literature.

Previous studies have reported health service differences by geographical area although they did not identify what systemic factors are responsible [19, 20]. Population density may account for readmission rates [19] but this is disputed by other studies [62–64]. In our study, we found that the readmission rate in Regina is lower than Saskatoon, which could be possibly due to the difference in population density in those areas. There could be some other underlying reasons as well, like distance to the nearest inpatient service, availability of community health services and factors that are likely to affect service use and aggregate service needs. However, Saskatoon patients are less likely to be hospitalized for mental conditions compared to Regina, Lloydminster and the rest of Saskatchewan. Further research is needed to explain this paradoxical result.

For outpatient psychiatric or general physician mental health care, our results indicate that visiting a general physician prior to the index date protects patients from having multiple hospitalizations. However, the logistic component shows that visiting a general physician in the two years prior to the index visit plays a protective role in case of hospitalization. One possible interpretation of these results could be that visits to a general physician may reflect a clinical assessment of lower risk or severity as compared with patients referred to acute services. Referral to more specialized services (e.g. FFS psychiatrist) also seems to increase the readmission risk. This may indicate that patients are not seen by psychiatrists until they are very seriously ill. It is assumed that people who are referred to a psychiatrist usually have a more serious condition that is better handled by a specialist in mental health, rather than a general physician. The association between visit to any FFS psychiatrist and higher admission rate could also indicate that those patients were in the psychiatric waiting list for sometime but as they had a severe issue, had to end up in a hospital. Psychosis accounts for 60 percent of mental health hospitalizations [65] as hospitals are better equipped to contain risk. However, the de-institutionalization movement in most developed countries has emphasized the need for greater community-based mental health care [66–68].

### Limitations

Our results are subject to some limitations. For this study, we could not consider some possible factors like the socio-economic status of the patients, their income level and sources, since the information was not captured in the administrative health databases. Linkage of the administrative databases used in the study to the survey data could provide the opportunity of investigating the influence of these potential explanatory variables on the psychiatric inpatient use. The other possible factor could be if the admitted patients were given psychiatric beds or not, since the unavailability of psychiatric beds can lead to a premature discharge for some patients and increases the risk for a future readmission. Ideally, it would be more natural for the 6-29 age group to be split into 6-18 and then 19-29 so as to conform to the division of child and adolescent psychiatry on one hand, and adult psychiatry on the other. The age group of 60-86 can also be split into a senior citizen and an elderly group served by geriatric psychiatrists. However, the sample sizes of the categories on the tails are small, which makes comparisons with the middle age groups very challenging. Future studies based on a large sample is warranted in order to investigate the age effect more properly. The registered Indian status variable was based on treaty status and does not include Metis and other aboriginal peoples without treaty status. The “others” mental disorder category consists of heterogeneous group of diseases like: eating disorder, sexual preference disorder. We used this categorization of disorders following a similar study among children by Rosychuk and colleagues [69]. We are not sure if these diseases are a homogeneous grouping.

In addition, easy access to mental health services could prevent some hospitalization for non-severe cases. As a result, the availability of outpatient and inpatient mental care at the area-level (e.g., number of doctor’s offices and the number of hospital beds per 100,000 inhabitants) may also influence psychiatric inpatient use beyond the effects of individual-level factors. However, we do not have this information in the current study. Future research incorporating such information is needed for understanding areal-level differences in the mental healthcare supplies that influence psychiatric inpatient use, which can assist in planning prevention efforts that are more tailored to the needs of a region.

## Conclusions

This study leads to a better understanding of factors contributing to increased inpatient hospitalizations among patients with mental health conditions, which may help health professionals to detect high risk populations for prevention. Patients with mental health conditions may benefit from greater and more timely access to primary care. Providing easier access to registered Indian people and youth may reduce the need for hospital-based care.

## Supplementary information

Additional file Supplementary Materials for “Modeling Socio-demographic and Clinical Factors Influencing Psychiatric Inpatient Service Use: A Comparison of Models for Zero-Inflated and Overdispersed Count Data”

## Data Availability

The data could be made available subject to approval from the Ministry of Health, Saskatchewan.

## Abbreviations

ICD: International Classification of Diseases
GP: General Physician
FFS: Fee-For-Service
NB: Negative Binomial
ZIP: Zero-Inflated Poisson
ZINB: Zero-Inflated Negative Binomial
HP: Hurdle Poisson
HNB: Hurdle Negative Binomial
AIC: Akaike’s Information Criterion
RQR: Randomized Quantile Residual
CDF: Cumulative Distribution Functions
CI: Confidence Interval
OR: Odds Ratio

## References

[1] Lyons JS, O’Mahoney MT, Miller SI, Neme J, Kabat J, Miller F (1997). Predicting readmission to the psychiatric hospital in a managed care environment: implications for quality indicators. Am J Psychiatr.

[2] Taube CA, Goldman HH, Burus BJ, Kessler LG (1988). High users of outpatient mental health services, I: Definition and characteristics. Am J Psychiatr.

[3] Nawaf M, Zhao H, Fang LJ (2007). Hospital readmissions for patients with mental illness in Canada. Healthc Q.

[4] De Oliveira C, Cheng J, Vigod S, Rehm J, Kurdyak P (2016). Patients with high mental health costs incur over 30 percent more costs than other high-cost patients. Health Aff.

[5] Machado V, Leonidas C, Santos MA, Souza J (2012). Psychiatric readmission: an integrative review of the literature. Int Nurs Rev.

[6] Durbin J, Lin E, Layne C, Teed M (2007). Is readmission a valid indicator of the quality of inpatient psychiatric care?. J Behav Health Serv Res.

[7] Rumball-Smith J, Hider P (2009). The validity of readmission rate as a marker of the quality of hospital care, and a recommendation for its definition. N Z Med J.

[8] Anderson M, Revie CW, Quail JM, Wodchis W, de Oliveira C, Osman M, Baetz M, McClure J, Stryhn H, Buckeridge D, Neudorf C (2018). The effect of socio-demographic factors on mental health and addiction high-cost use: a retrospective, population-based study in Saskatchewan. Can J Public Health.

[9] Kalseth J, Lassemo E, Wahlbeck K, Haaramo P, Magnussen J. Psychiatric readmissions and their association with environmental and health system characteristics: a systematic review of the literature. BMC Psychiatry. 2016; 16(376). 10.1186/s12888-016-1099-8 PMC5100223 27821155

[10] Appleby L, Luchins DJ, Freels S, Smith ME, Wasmer D (2008). The impact of immigration on psychiatric hospitalization in Illinois from 1993 to 2003. Psychiatr Serv.

[11] Klinkenberg WD, Calsyn RJ (1996). Predictors of receipt of aftercare and recidivism among persons with severe mental illness: a review. Psychiatr Serv.

[12] Lin C-H, Chen M-C, Chou L-S, Lin C-H, Chen C-C, Lane H-Y (2010). Time to rehospitalization in patients with major depression vs. those with schizophrenia or bipolar I disorder in a public psychiatric hospital. Psychiatry Res.

[13] Weiden P, Aquila R, Standard J (1996). Atypical antipsychotic drugs and long-term outcome in schizophrenia. J Clin Psychiatry.

[14] John LH, Offord DR, Boyle MH, Racine YA (1995). Factors predicting use of mental health and social services by children 6–16 years old: Findings from the Ontario Child Health Study. Am J Orthopsychiatry.

[15] Palin JL, Goldner EM, Koehoorn M, Hertzman C (2011). Primary mental health care visits in self-reported data versus provincial administrative records. Health Rep.

[16] Waddell C, Offord DR, Shepherd CA, Hua JM, McEwan K (2002). Child psychiatric epidemiology and Canadian public policy-making: The state of the science and the art of the possible. Can J Psychiatr.

[17] Offord DR, Boyle MH, Fleming JE, Blum HM, Rae Grant NI (1989). Summary of selected results. Can J Psychiatr.

[18] Carrière GM, Bougie E, Kohen D, Rotermann M, Sanmartin C (2016). Acute Care Hospitalization by Aboriginal Identity, Canada, 2006 Through 2008. Health Rep.

[19] Rüesch P, Meyer P, Hell D (2000). Who is rehospitalized in a psychiatric hospital? Psychiatric hospitalization rates and social indicators in the Zurich canton (Switzerland). Gesundheitswesen (Bundesverband der Arzte des Offentlichen Gesundheitsdienstes (Germany)).

[20] Lin CH, Chen WL, Lin CM, Lee RN, Ko MC, Li CY (2010). Predictors of psychiatric readmissions in the short-and long-term: a population-based study in Taiwan. Clinics.

[21] Priebe S, Katsakou C, Amos T, Leese M, Morriss R, Rose D, Wykes T, Yeeles K (2009). Patients’ views and readmissions 1 year after involuntary hospitalisation. Br J Psychiatr.

[22] Brennan PL, Kagay CR, Geppert JJ, Moos RH (2000). Elderly Medicare inpatients with substance use disorders: characteristics and predictors of hospital readmissions over a four-year interval. J Stud Alcohol.

[23] Carrière GM, Bougie E, Kohen D, Rotermann M, Sanmartin C (2016). Acute Care Hospitalization by Aboriginal Identity, Canada, 2006 Through 2008. Health Reports.

[24] Ono T, Tamai A, Takeuchi D, Tamai Y (2011). Factors related to readmission to a ward for dementia patients: Sex differences. Psychiatry Clin Neurosci.

[25] Craig TJ, Fennig S, Tanenberg-Karant M, Bromet EJ (2000). Rapid versus delayed readmission in first-admission psychosis: quality indicators for managed care?. Ann Clin Psychiatry.

[26] Morrow-Howell N, Proctor E, Blinne W, Rubin E, Saunders J, Rozario P (2006). Post-acute dispositions of older adults hospitalized for depression. Aging Ment Health.

[27] Houston S (2002). Steps on the Road to Medicare: Why Saskatchewan Led the Way.

[28] eHealth Saskatchewan. Eligibility for Health Benefits. 2017. Retrieved from https://www.ehealthsask.ca/residents/health-cards/Pages/Eligibility-for-Health-Benefits.aspx.

[29] Lyon M, Lyon N, Magnusson MS (1994). The importance of temporal structure in analyzing schizophrenic behavior: some theoretical and diagnostic implications. Schizophr Res.

[30] Morgan V, Castle D, Page A, Fazio S, Gurrin L, Burton P, Montgomery P, Jablensky A (1997). Influenza epidemics and incidence of schizophrenia, affective disorders and mental retardation in Western Australia: no evidence of a major effect. Schizophr Res.

[31] Selten J-P, Slaets J, Kahn RS (1997). Schizophrenia in Surinamese and Dutch Antillean immigrants to The Netherlands: evidence of an increased incidence. Psychol Med.

[32] Tansella M, Micciolo R, Biggeri A, Bisoffi G, Balestrieri M (1995). Episodes of care for first-ever psychiatric patients a long-term case-register evaluation in a mainly urban area. Br J Psychiatr.

[33] Forbes C, Evans M, Hastings N, Peacock B (2011). Statistical Distributions.

[34] Johnson NL, Kemp AW, Kotz S (2005). Univariate Discrete Distributions.

[35] Selvin S (1995). Practical Biostatistical Methods.

[36] McKenzie DP, Mackinnon AJ, Martindale C, Clarke DM (1998). Modelling the frequency of psychiatric admissions. Int J Methods Psychiatr Res.

[37] Gardner W, Mulvey EP, Shaw EC (1995). Regression analyses of counts and rates: Poisson, overdispersed Poisson, and negative binomial models. Psychol Bull.

[38] van den Broek J (1995). A score test for zero inflation in a Poisson distribution. Biometrics.

[39] Mullahy J (1986). Specification and testing of some modified count data models. J Econom.

[40] Zuur AF, Leno EN, Walker NJ, Saveliev AA, Smith GM (2009). Zero-truncated and zero-inflated models for count data. Mixed Effects Models and Extensions in Ecology with R.

[41] Akaike H, Petrov BN, Csaki F (1973). Information theory and an extension of the maximum likelihood principle. Second international symposium on information theory.

[42] Vuong QH (1989). Likelihood ratio tests for model selection and non-nested hypotheses. Econometrica.

[43] Dunn PK, Smyth GK (1996). Randomized quantile residuals. J Comput Graph Stat.

[44] Feng C, Li L, Sadeghpour A (2020). A comparison of residual diagnosis tools for diagnosing regression models for count data. BMC Med Res Methodol.

[45] Xia YL, Morrison-Beedy D, Ma JM, Feng GY, Cross W, Tu X (2012). Modeling count outcomes from HIV risk reduction interventions: A comparison of competing statistical models for count responses. AIDS Res Treat.

[46] Xu L, Paterson AD, Turpin W, Xu W (2015). Assessment and selection of competing models for zero-inflated microbiome data. PLoS ONE.

[47] Brooks ME, Kristensen K, van Benthem KJ, Magnusson A, Berg CW, Nielsen A, Skaug HJ, Maechler M, Bolker BM (2017). glmmTMB balances speed and flexibility among packages for zero-inflated generalized linear mixed modeling. R Journal.

[48] Adelson N (2005). The embodiment of inequity: Health disparities in Aboriginal Canada. Can J Public Health.

[49] Bombay A, Matheson K, Anisman H (2009). Intergenerational trauma. J Santé autochtone.

[50] Browne AJ, Smye VL, Rodney P, Tang SY, Mussell B, O’Neil J (2011). Access to primary care from the perspective of Aboriginal patients at an urban emergency department. Qual Health Res.

[51] Firestone M, Smylie J, Maracle S, McKnight C, Spiller M, O’Campo P (2015). Mental health and substance use in an urban First Nations population in Hamilton, Ontario. Can J Public Health.

[52] Allan B, Smylie J (2015). First Peoples, Second Class Treatment: The Role of Racism in the Health and Well-being of Indigenous Peoples in Canada, Discussion Paper.

[53] Bingham B, Jebamani L, Bonshor L, OŠNeil J, Edel K, Paul H, George J, Laing K, Bird J, Bartz T. Aboriginal community-based primary health care research: Developing community-driven primary health care research priorities. Surrey, British Columbia: Aboriginal Health Services, Fraser Health. 2013.

[54] Merikangas KR, Nakamura EF, Kessler RC (2009). Epidemiology of mental disorders in children and adolescents. Dialogues Clin Neurosci.

[55] Costello EJ, Mustillo S, Erkanli A, Keeler G, Angold A (2003). Prevalence and development of psychiatric disorders in childhood and adolescence. Arch Gen Psychiatr.

[56] Costello EJ, Egger H, Angold A (2005). 10-year research update review: the epidemiology of child and adolescent psychiatric disorders: I, Methods and public health burden. J Am Acad Child Adolesc Psychiatry.

[57] Brauner CB, Stephens CB (2006). Estimating the prevalence of early childhood serious emotional/behavioral disorders: Challenges and recommendations. Public Health Rep.

[58] World Health Organization. Gender and women’s mental health. 2020. Retrieved from https://www.who.int/mental_health/prevention/genderwomen/en/.

[59] McLean CP, Asnaani A, Litz BT, Hofmann SG (2011). Gender differences in anxiety disorders: prevalence, course of illness, comorbidity and burden of illness. J Psychiatr Res.

[60] Hawton K (2000). Sex and suicide: Gender differences in suicidal behaviour. Br J Psychiatr.

[61] Maguen S, Cohen B, Cohen G, Madden E, Bertenthal D, Seal K (2012). Gender differences in health service utilization among Iraq and Afghanistan veterans with posttraumatic stress disorder. J Women’s Health.

[62] Prince JD, Akincigil A, Kalay E, Walkup JT, Hoover DR, Lucas J, Bowblis J, Crystal S (2008). Psychiatric rehospitalization among elderly persons in the United States. Psychiatr Serv.

[63] Husted J, Jorgens A (2000). Best practices: population density as a factor in the rehospitalization of persons with serious and persistent mental illness. Psychiatr Serv.

[64] Stahler GJ, Mennis J, Cotlar R, Baron DA (2009). The influence of neighborhood environment on treatment continuity and rehospitalization in dually diagnosed patients discharged from acute inpatient care. Am J Psychiatry.

[65] Zhang J, Harvey C, Andrew C (2011). Factors associated with length of stay and the risk of readmission in an acute psychiatric inpatient facility: a retrospective study. Aust N Z J Psychiatr.

[66] Beecham J (2005). Access to mental health supports in England: crisis resolution teams and day services. Int J Law Psychiatry.

[67] Gaddini A, Ascoli M, Biscaglia L (2005). Mental health care in Rome. Eur Psychiatr.

[68] Guo S, Biegel DE, Johnsen JA, Dyches H (2001). Assessing the impact of community-based mobile crisis services on preventing hospitalization. Psychiatr Serv.

[69] Newton AS, Rathee S, Grewal S, Dow N, Rosychuk RJ (2014). Children’s mental health visits to the emergency department: factors affecting wait times and length of stay. Emerg Med Int.

