# Supplementary material for: Modeling socio-demographic and clinical factors influencing psychiatric inpatient service use: a comparison of models for zero-Inflated and overdispersed count data
Source: BMC Med Res Methodol. 2020 Sep 16;20:232. doi: 10.1186/s12874-020-01112-w (PMC7495888; doi:10.1186/s12874-020-01112-w)
Supplement: Supplementary file 1 — Supplementary Materials for “Modeling Socio-demographic and Clinical Factors Influencing Psychiatric Inpatient Service Use: A Comparison of Models for Zero-Inflated and Overdispersed Count Data” [file 12874_2020_1112_MOESM1_ESM.pdf]

Supplementary Materials for "Modeling Socio-demographic and Clinical Factors Influencing Psychiatric Inpatient Service Use: A Comparison of Models for Zero-Inflated and Overdispersed Count Data"

August 1, 2020

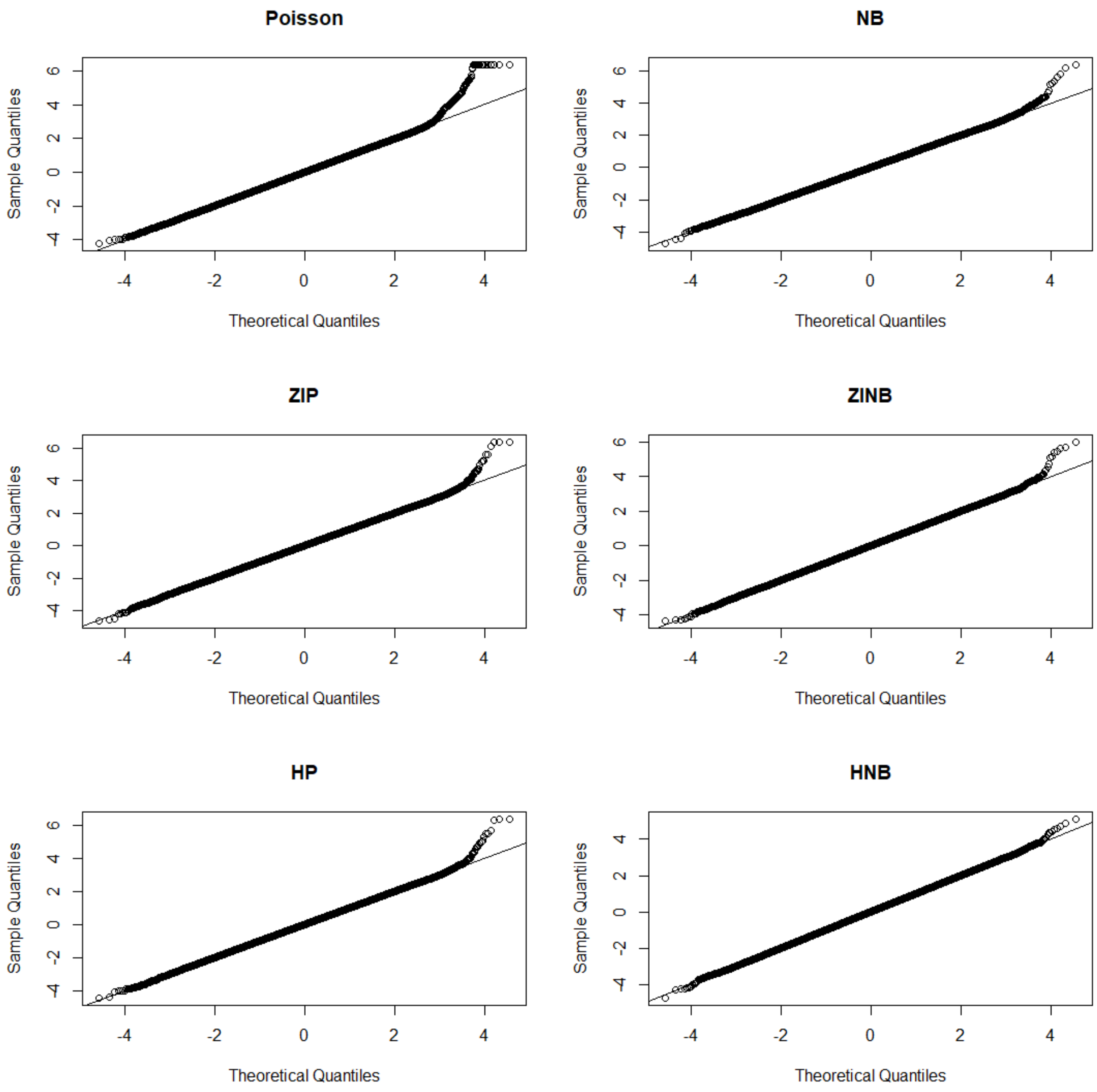

Figure S1: QQ normality plots of RQRs for all the competing models for the 3 months follow-up study period.

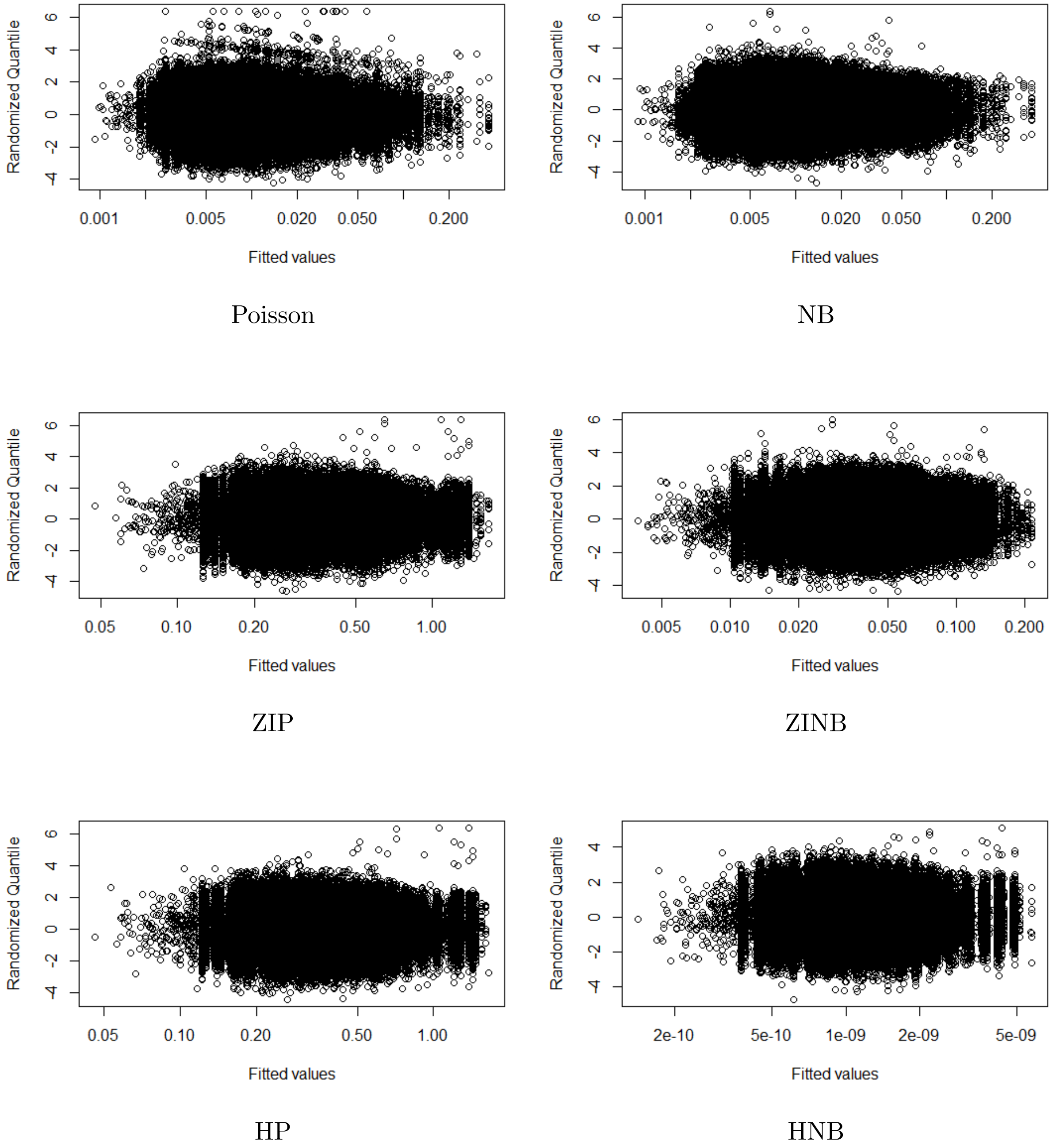

Figure S2: Scatter plots of RQRs for all the competing models for the 3 months follow-up study period.

Table S1: Observed vs. Predicted frequencies of the number of repeated inpatient hospitalizations for the 3 months follow-up period.

	Observed	Predicted frequencies					
Unique y	Frequencies	HNB	HP	ZINB	ZIP	NB	Poisson
0	199271	199271	199271	199279.3	199274.4	199272.2	199064
1	1124	1100.11	1077.46	1076.34	1068.52	1094.99	1460.30
2	118	131.06	159.92	143.05	164.14	126.14	12.52
3	10	25.28	23.92	28.01	25.08	27.36	0.20
4	1	6.41	3.88	7.02	4.04	8.72	0
5	2	1.96	0.67	2.08	0.67	3.54	0
6	4	0.68	0.11	0.70	0.11	1.68	0
7	2	0.26	0.01	0.25	0.01	0.88	0
8	2	0.11	0	0.10	0	0.05	0
9	2	0.04	0	0.04	0	0.03	0
10	1	0.02	0	0	0	0.01	0
